# Rescue Procedure in a Rare Case of Iatrogenic Vertebral Artery Puncture and Review of the Literature

**DOI:** 10.3390/jcm14196945

**Published:** 2025-09-30

**Authors:** Jonas Brandt, Manfred Musigmann, Burak Han Akkurt, Michael Köhler, Hermann Krähling

**Affiliations:** 1 University Clinic for Radiology, University of Münster, University Hospital Münster, Albert-Schweitzer-Campus 1, 48149 Münster, Germany; 2 University Clinic for Radiology, Department for Interventional Neuroradiology, University of Münster, University Hospital Münster, Albert-Schweitzer-Campus 1, 48149 Münster, Germany

**Keywords:** iatrogenic vessel perforation, endovascular treatment, neuroradiological intervention, supra-aortic stenting, BeGraft Stents

## Abstract

**Objective:** The insertion of venous catheters into supra-aortic vessels is a standard procedure in the treatment of a large number of diseases. Incorrect placement of venous catheters into and accidental perforation of arterial vessels is a rare but serious complication that can lead to severe bleeding, dissections, and strokes. **Materials and Methods:** We present a case of iatrogenic malposition of a Broviac catheter into the right vertebral artery and its successful treatment through stenting of the perforation site using a Walrus balloon catheter to achieve a secure treatment position and eventually cover the perforation site. Additionally, we conducted a systematic literature review on endovascular rescue procedures in inadvertent injuries of the supra-aortic arteries related to venous catheter placement. **Results:** A balloon guide catheter was used to achieve a secure treatment position for the deployment of a stent graft covering the perforation site, ensuring no significant arterial hemorrhage occurred during the removal of the incorrectly placed Broviac catheter. The review of the literature on endovascular rescue procedures for supra-aortic arterial vessel damage caused by venous catheter placement revealed that endovascular treatment has been successful in all reported cases of catheter-related supra-aortic arterial injury. Primary stent graft placement without additional actions was the most common treatment approach. **Conclusions:** In the treatment of iatrogenic injuries to the supra-aortic vessels, endovascular treatment strategies represent a safe, reliable, and generally recognized option and thus are used much more frequently than surgical procedures. **Key point:** In rare cases of accidental malposition of venous catheters into supra-aortic arterial vessels, endovascular insertion of a stent graft covering the perforation site using a balloon guide catheter can be a safe treatment option.

## 1. Introduction

Annually, over five million central venous catheters (CVCs) are placed in the United States of America, including Broviac catheters, special venous accesses that are used in particular in oncology and intensive care medicine for the long-term treatment of patients [1]. The Broviac catheter was developed in 1973 by the American surgeon Dr John Broviac [2]. It is a central venous catheter that establishes a direct connection to large veins, usually the superior vena cava, internal jugular vein, or subclavian vein [3]. The silicone or polyurethane catheter often consists of a single or double lumen tube through which medication, fluid, or nutrients can be administered directly into the patient’s bloodstream. The particular advantage of the Broviac catheter is the potentially long retention time in the patient’s body (weeks to months) [4,5]. Typical complications associated with the use of Broviac catheters are primarily the occurrence of catheter-associated infections, but also catheter thrombosis or dislocation of the catheter [6]. Incorrect placement of the Broviac catheter is rare, but usually leads to serious complications [7]. Arterial malposition of CVCs, in general, does not occur infrequently, with published rates between 3 and 12%, mainly involving the carotid arteries and leading to increased hospital stay, morbidity, mortality, and healthcare costs [8,9].

In the following sections, we present a rare case of iatrogenic malposition of a Broviac catheter into the right vertebral artery and its successful endovascular treatment.

There are three main treatment approaches of such injuries, including pressure hemostasis, surgical repair, and endovascular treatment [10]. While pressure hemostasis may have limited effect due to unfavorable location of the injury site [1,10] and surgical repair can be associated with a high degree of morbidity in usually critically ill patients, endovascular treatment is increasingly performed in these cases [8].

Regarding endovascular treatment, there are several options, including ballon occlusion, stent implantation, embolization (e.g., with detachable coils), and use of percutaneous closure devices [1,8,10].

The case of iatrogenic injury of the right vertebral artery during the placement of a Broviac catheter in our tertiary hospital presented in the following sections prompted a systematic literature search, which revealed that reports of acute endovascular treatment of inadvertent injury of supra-aortic arteries are rare and currently limited to case reports [10]. At the same time, there are no treatment guidelines for therapy of such injuries [10]. We therefore aimed to create this comprehensive review, illustrating endovascular treatment approaches for acute inadvertent injury of supra-aortic arteries with CVCs, to elucidate which treatment strategies seem feasible.

## 2. Systematic Review

No approval by the institutional review board was needed for this literature-based review.

**Figure 1:**
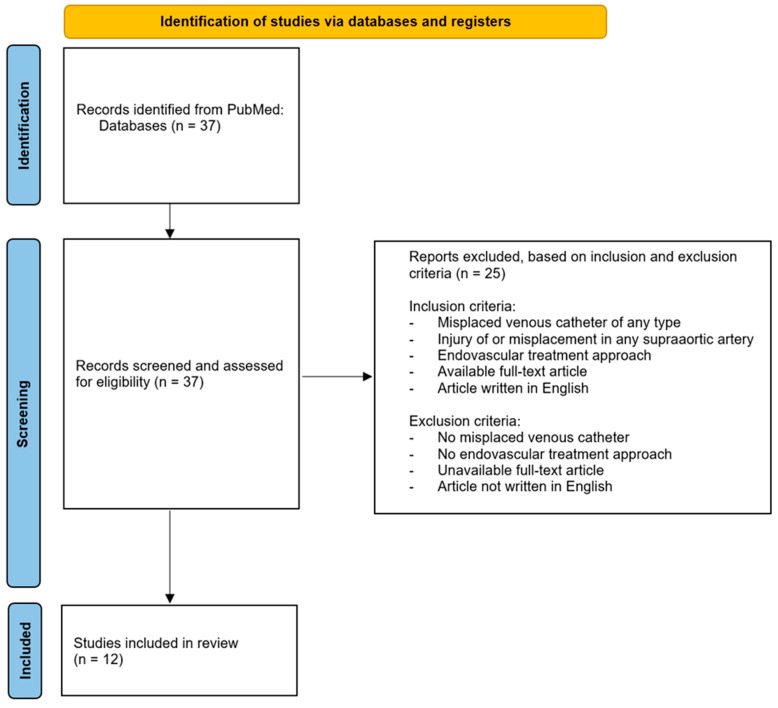
Flow chart illustrating search strategy, in- and exclusion criteria, and included studies for this systematic review, based on the PRISMA guidelines for reporting systematic reviews.

We conducted a systematic review of the medical literature to identify published cases of inadvertent injury of supra-aortic arteries related to venous catheter placement using the online databases of MED-LINE/PubMed between introduction and February 2024. The search terms were “misplaced venous catheter” OR “misplaced broviac” OR “misplaced shaldon” OR “misplaced central venous catheter” AND “stroke” OR “cerebral angiography” OR “balloon guided catheter” OR “ischemia” OR “stent” OR “rescue”. Inclusion criteria were (1) misplaced venous catheters of any type, (2) injury of or misplacement in any supra-aortic artery, (3) endovascular treatment approach, (4) available full-text articles, and (5) articles written in English. Exclusion criteria were (1) no endovascular treatment approach, (2) no misplaced venous catheter, (3) unavailable full-text articles, and (4) articles written in languages other than English (Figure 1). We conducted a review of all articles that met our inclusion and exclusion criteria. Senior radiology residents with three to nine years of experience in diagnostic neuroradiology independently screened titles and/or abstracts of the studies retrieved by using the aforementioned systematic search plan and extracted data from eligible studies into a standardized form. Discrepancies in the evaluation of the studies were resolved by consensus or by consultation with a senior reviewer.

Initial search according to our search strategy showed 37 studies (Table 1 and Table 2). Thorough analysis of the available material of each article lead to the exclusion of 25 studies due to a missed topic or not meeting the inclusion criteria. Our search revealed 12 studies that described inadvertent injury of supra-aortic arteries related to venous catheter placement that were treated using an endovascular approach [1,7,8,9,10,11,12,13,14,15,16,17]. In detail, we identified two retrospective studies [7,10], and the remaining publications were case reports. Seven publications described injuries solely of the subclavian artery [1,7,8,10,12,13,16], three publications described injuries solely of the carotid artery [9,15,17], and two case reports described injuries of the vertebral artery [11,14].

In total, the review of the current literature showed 16 cases of inadvertent injury of supra-aortic arteries related to venous catheter placement that were treated using an endovascular approach. Table 1 and Table 2 provide a detailed summary of the information extracted from the included publications, including source references.

Catheters involved were central venous catheters (*n* = 11; 69%), catheters for hemodialysis (*n* = 2; 13%), one Swan–Ganz catheter (6%), one port catheter (6%), and one sheath (6%). Supra-aortic arteries injured were the right subclavian artery (RSCA; *n* = 10; 63%), right common carotid artery (RACC; *n* = 3; 19%), right vertebral artery (RAVT; *n* = 2; 13%), and left subclavian artery (LSCA; *n* = 1; 6%).

Seven patients (44%) did not show any symptoms related to arterial injury. Two patients (13%) presented with solely signs of hemorrhage. Two patients (13%) showed signs of hemorrhage and additionally developed a pseudoaneurysm (of the RACC and RSCA, respectively). One patient (6%) presented solely with an AV fistula between the RACC and the right jugular vein. One patient (6%) showed an occlusion of the RAVT due to a misplaced catheter but no symptoms of stroke due to a good crossflow from the left vertebral artery. One patient (6%) showed hemorrhage and symptoms of stroke in the territory of the RAVT and right posterior cerebral artery due to injury of the RSCA. One patient (6%) showed solely symptoms of stroke in the territory of the right anterior and medial cerebral artery due to a misplaced catheter in the RACC.

Endovascular approaches were used in all cases. Positioning of a stent graft over the entry point of the misplaced catheter was the most common treatment approach, being used in 14 cases (88%). In detail, stenting was used in 10 cases (71%) as the only treatment approach. Stenting was combined with manual compression of the entry point of the misplaced catheter in three cases (29%). In one case (6%), stenting was combined with temporary balloon occlusion. A combination of percutaneous closure and temporary balloon occlusion was applied in one case (6%). Coiling was performed in one case (6%) as the only treatment approach. In detail, the injured RAVT was embolized after performing MRI perfusion studies, showing sufficient crossflow from the left vertebral artery [11].

All approaches were technically successful and conversion to open surgery was not necessary in any case. Periinterventional complications occurred in two cases (13%). One patient (6%) suffered from a right-sided cerebral infarction after repair of the RSCA with a stent graft, most likely caused by retrograde embolism during the procedure. One patient (6%) developed a small pseudoaneurysm. Follow-up angiography after six weeks demonstrated a complete spontaneous resolution of the pseudoaneurysm. All outcomes were rated favorable.

## 3. Clinical Case

**Figure 2:**
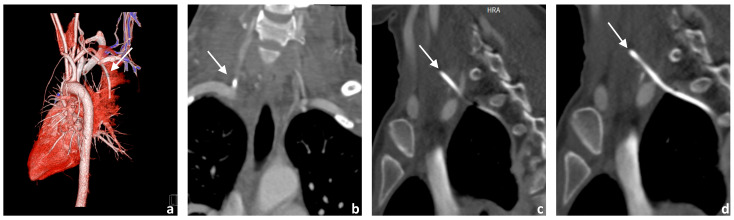
Imaging findings of the patient at initial presentation (**a**–**d**): 3D reconstruction of the CT angiography and supra-aortic vessels (**a**); CT angiography reformats of the course of the right vertebral artery in coronary (**b**) and sagittal orientation (**c**,**d**). The course of the incorrectly inserted Broviac catheter is indicated by arrows. The Broviac catheter inserted incorrectly into the V0 or V1 segment of the right vertebral artery ends with its tip in the pleural cavity.

A patient in her 20s was presented to our interventional radiology section at our specialized tertiary hospital following the sonographically guided placement of a Broviac catheter. The placement of the Broviac catheter initially seemed to be without any major complications, and only difficult aspiration via the catheter was detected. This prompted a recheck using fluoroscopy. The tip of the catheter projected on to the subclavian vein, leading to repositioning under fluoroscopy without applying contrast agent during this process. Even in the new position, the catheter could be flushed normally with a heparin solution, but aspiration was still very difficult. A CT angiography confirmed malposition of the Broviac catheter outside the venous system with perforation of the right vertebral artery and localization of the tip in the pleural cavity dorsal of the vena cava superior (Figure 2).

At this point, an interdisciplinary decision between the departments of vascular surgery and interventional neuroradiology was made on interventional salvage of the catheter and covering the perforation site with a stent (see Section 2 and Section 3).

### 3.1. Image Acquisition

Preinterventional cerebral imaging, including non-contrast cerebral computed tomography, CT angiography and CT perfusion, was performed to rule out the exact position of the catheter, visualize the vascular anatomy, and exclude other major complications of the cervical vasculature as well as cerebral perfusion damage.

Scans were performed on single- and dual-source CT scanners (SOMATOM Force, 2 × 192 × 0.24 mm dual energy acquisition; SOMATOM Definition AS+, 64 × 0.6 mm × 2 alternating focal spot slice acquisition; all scanners from Siemens Healthcare) with patient-based tube current modulation. In total, 80 cc of iodinated contrast agent (Ultravist 370, Bayer Healthcare, Berlin, Germany) was injected with a flow rate of 4 cc/s using a power injector. Image reconstruction was performed with 0.6–1.0 mm slice thickness for multiplanar reformation.

The examination protocol consisted of a non-contrast head CT scan followed by a CT angiography of the supra-aortic and cerebral vasculature (80 cc contrast medium; 4 cc/s flow rate) and a CT perfusion study of the head (30 cc contrast medium; 5 cc/s flow rate) while the patients’ arms were lowered.

### 3.2. Neurointerventional Procedure

Diagnostic angiography of the cerebral and supra-aortic arteries was performed via an arterial transfemoral approach in general anesthesia.

A selective catheter angiography of the right vertebral artery was first performed to rule out a PICA ending before planned catheter removal.

**Figure 3:**
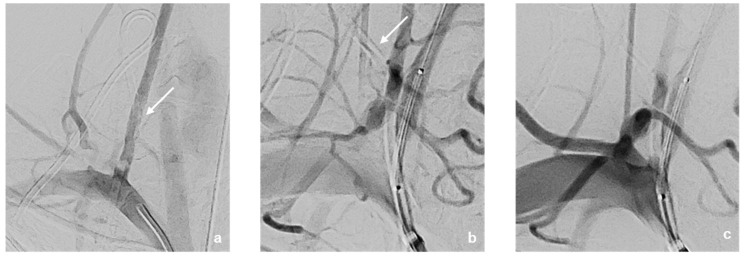
DSA—Imaging findings of the patient at initial presentation (**a**–**c**). The course of the incorrectly inserted Broviac catheter is indicated by arrows. The Broviac catheter inserted incorrectly into the V0-/V1-segment of the right vertebral artery (**a**) ends with its tip in the pleural cavity. A BeGraft stent (4.5 mm × 21 mm) was placed directly at the proximal outlet of the right vertebral artery (**b**,**c**).

**Figure 4:**
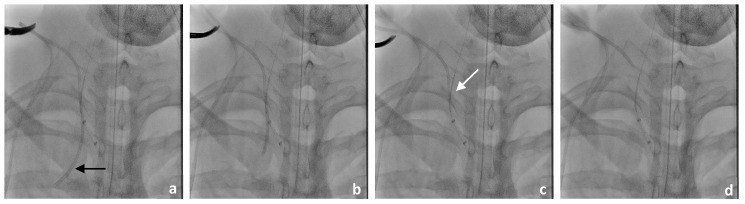
(**a**–**d**): Step-by-step careful manual retrieval of the Broviac catheter (indicated by a black arrow) under fluoroscopic control. A BeGraft stent (4.5 mm × 21 mm) was placed directly at the proximal outlet of the right vertebral artery (indicated by a white arrow).

**Figure 5:**
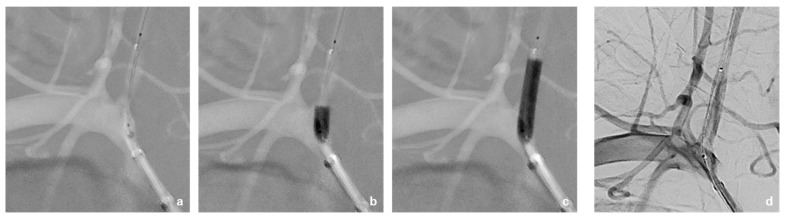
Implantation of the BeGraft stent at the perforation site. (**a**): The stent had already been placed in the right vertebral artery. (**b**,**c**): Deployment of the stent with the help of a premounted balloon without technical complications. (**d**): After release of the BeGraft stent, there was good contrast of the normal caliber vascular lumen of the right vertebral artery and there was no flow-limiting damage to the vessel wall.

This was followed by selective probing of the right vertebral artery using an 8F Walrus balloon catheter guided via a pORTAL 0.014′ microwire (Figure 3). After checking the correct catheter/wire position, a BeGraft stent (4.5 mm × 21 mm) was positioned but not yet released at the perforation site directly at the proximal outlet of the right vertebral artery (Figure 3b,c). This was followed by careful manual retrieval of the Broviac catheter under fluoroscopic control (Figure 4). After the removal of the Broviac catheter, vascular wall irregularities of the vertebral artery were seen with simultaneous arterial bleeding from the cutaneous access site of the Broviac catheter. Therefore, the indication for implantation of the BeGraft stent was given, which had already been placed in the vertebral artery and could be deployed without technical complications (Figure 5).

After the release of the BeGraft stent, there was good contrast of the normal caliber vascular lumen of the right vertebral artery and there were no flow-limiting endoluminal vascular changes (Figure 4d). There was no peripheral vascular occlusion due to a thrombus in the supply area of the right vertebral artery.

The following materials were used as standard for diagnostic angiography and stent implantation in the patient:

8F Cook sheath (Cook Medical, Bloomington, IN, USA), Terumo standard and stiff wire (Radiofocus, Tokyo, Japan), pORTALmicrowire 0.014 inch (Phenox, Bochum, Germany), Walrus balloon guide catheter (Q’Apel Medical, Fremonnt, CA, USA), BeGraft Coronary Stent Graft System 4.5 × 21 mm (Bentley InnoMed, Hechingen, Germany).

### 3.3. Drug Therapy During Intervention and After Stent Implantation

#### 3.3.1. Pre-Interventional Drug Administration

Clopidogrel 75 mg (1-0-0/d) for five days.

#### 3.3.2. Periinterventional Drug Administration

500 mg ASA + Eptifibatid bolus and infusion (body weight adapted) for 24 h.

#### 3.3.3. Postinterventional Drug Administration

Loading with Clopidogrel 300 mg (1-0-0/d), 24 h later start of a double platelet aggregation inhibition with Clopidogrel 75 mg (1-0-0/d) + 100 mg ASA (1-0-0/d) for twelve months, followed by a mono platelet aggregation inhibition with 100 mg ASA (1-0-0/d) life-long.

### 3.4. Postinterventional Result

Using a balloon guide catheter, a safe treatment position for the release of a stent graft covering the perforation site could be achieved without a significant arterial hemorrhage occurring during the removal of the incorrectly inserted Broviac catheter.

The vessel remodeling effect of the implanted stent and the DSA imaging of patient are given in Figure 5d.

**Figure 6:**
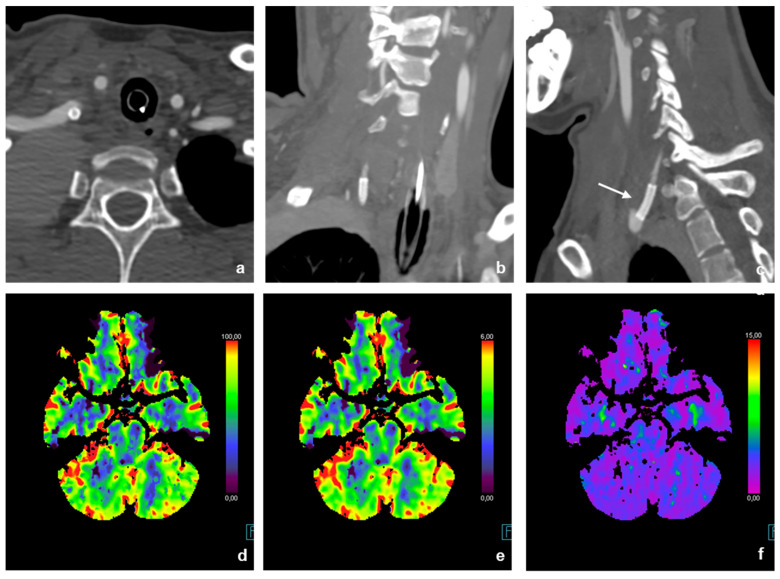
Postinterventional CT-A and CT-P. Correctly inserted BeGraft Stent in the V0-Segment of the right vertebral artery ((**a**–**c**), indicated by a white arrow). The postinterventional cerebral CT–P controls showed no focal ischemia (**d**–**f**).

The postinterventional cerebral CT controls showed no focal ischemia (Figure 6d–f), and the neurological status of the patient showed no marked clinical deterioration.

## 4. Discussion

Our analysis of the available literature showed that catheter-related supra-aortic arterial injury is a rare but severe complication of percutaneous venous catheterization. Injuries were mainly caused using central venous catheters and affected the subclavian or carotid artery in the majority of cases [1,7,8,10,12,13,15,16,17,18]. Injuries of the RAVT were uncommon and described in only two cases [11,14]. Inadvertent arterial cannulation was asymptomatic in approximately 45% of all cases. Complications of misplaced catheters were severe, including hemorrhage, cerebral ischemia, pseudoaneurysm, and AV fistula [7,8,9,10,11,14,15,17].

Our review of the literature illustrates that endovascular treatment of catheter-related supra-aortic arterial injury was successful in all published cases [1,7,8,9,10,11,12,13,14,15,16,17,18]. Primary stent graft placement without additional actions was the most common treatment approach [1,9,12,13,16]. Additional manual compression of the entry site of the misplaced catheter seems to be beneficial in reducing the risk of hemorrhage [14,17] but was not possible in all cases due to the anatomical location of the injury. Using a balloon-guided catheter for stent placement seems to be an elegant approach as inflation of the balloon enables to reduce blood loss in cases of hemorrhage after removing the misplaced catheter and stabilizes the guidance catheter at the site of intervention. Covered stents show a good potential for securely covering the defect in the vessel wall at the site of entry of the misplaced catheter and good patency when anticoagulation is administered using established regimes [1,9,12,13,16]. Percutaneous closure was only described in one case, was not successful initially, and required several courses of additional balloon occlusion [8]. Coil embolization was performed successfully in one case, but is associated with a potential risk of ischemia and stroke, which seems unfavorable [11].

Complications of endovascular treatment were rare and of varying degree, including periinterventional stroke in one case [10] and a small pseudoaneurysm, both of which resolved spontaneously [8].

### Limitations and Future Directions

This review has several limitations: 1. This review is of a descriptive nature due to the lack of retrospective and prospective studies on endovascular treatment approaches for acute injuries of supra-aortic arteries in the context of CVC placement. This limitation has to be overcome in the future by carrying out such studies (preferably with a prospective approach) and by performing meta-analyses, if a sufficient grade of evidence is reached. 2. There might be a publication bias, caused by the publication of mainly successful endovascular interventions that might lead to the assumption that endovascular treatment of such injuries is extremely successful and safe. 3. We focused our review on endovascular treatment approaches of inadvertent supra-aortic artery injury during CVC placement, so that a direct comparison to pressure hemostasis and surgical repair is not possible.

## 5. Conclusions

In the treatment of iatrogenic injuries to the supra-aortic vessels, endovascular treatment strategies represent a safe, reliable, and generally recognized option with high technical success rates and thus might be a favorable alternative compared to surgical approaches.

## Figures and Tables

**Table 1:** Search terms and retrieved publications from PubMed, including author, year of publication, DOI, inclusion or exclusion status for our systematic review, reason for exclusion (if applicable), study type, and injured vessel.

Search Term	Title	Author	Year	doi	Included	Excluded	Unavailable Full Text	Language Other Than English	No Endovascular Approach	No Misplaced Catheter	Study Type	Carotid Placement	Vertebralis Placement	Subclavian Placement
misplaced venous catheter AND stroke	Arterial trauma during central venous catheter insertion: Case series, review and proposed algorithm	Guilbert et al.	2008	10.1016/j.jvs.2008.04.046		X			X		Review	X		X
	A rare cause of stroke: fail-implanted venous port catheter system - a case report		2021			X			X		Case report			
	Inadvertent subclavian artery catheter placement complicated by stroke: endovascular management and review	Jahromi et al.	2009	10.1002/ccd.21884	X						Case report			X
	Central Venous Catheter Placement Gone Awry: A Case Report of Right Internal Jugular Central Line Entering Subclavian Artery		2022			X			X		Case report			
	Misplaced peripherally inserted central catheter: an unusual cause of stroke		2004			X			X		Case report			
	Amidst COVID-19 pandemic: the catastrophic sequelae of an inadvertent carotid artery insertion during central venous catheter placement-a case report	Din et al.	2021	10.1080/20009666.2021.1952015	X						Case report	X		
	Thrombotic, infectious, and procedural complications of the jugular bulb catheter in the intensive care unit		1997			X				X	Clinical study			
	Right vertebral artery injury as a result of misplaced internal jugular vein catheter withdrawal		2019			X			X		Case report			
	Arterial misplacement of large-caliber cannulas during jugular vein catheterization: case for surgical management		2004			X			X		Clinical study			
	Truncus bicaroticus and an aberrant right subclavian artery contributing to internal jugular venous line misplacement into the carotid artery		2009			X			X		Case report			
	Use of echocardiography to identify appropriate placement of a central venous catheter wire in the vena cava prior to cannulation		2014			X			X	X	Review			
	Embolic strokes after peripherally inserted central catheter placement		2010			X			X		Case report			
misplaced venous catheter AND cerebral angiography	Elective and emergent endovascular treatment of subclavian artery aneurysms and injuries	Schoder et al.	2003	10.1177/152660280301000113	X						Retrospective study			X
misplaced venous catheter AND balloon guided catheter	Acute Endovascular Therapy for Iatrogenic Vertebral Artery Injury: A Case Report	Ito et al.	2021	10.5797/jnet.cr.2020-0208	X						Case report		X	
misplaced venous catheter AND ischemia	Inadvertent Arterial Cannulation and Norepinephrine Infusion Due to a Misplaced Central Venous Catheter		2021			X					Case report			
	Vascular anomaly diagnosis by central venous catheter misplacement: a case report		2022			X					Case report			
	Inadvertent catheter misplacement into the subclavian artery during ultrasound-guided internal jugular venous catheterization: a case report	Kohyama et al.	2023	10.1186/s40981-023-00649-1	X						Case report			X
	An atypical misplacement of a temporary pacing catheter diagnosed and resolved by ultrasound		2014			X			X	X	Case report			
	[Right subclavian catheterization. Misplaced insertion into the subclavian artery and the ascending aorta]		1999			X			X		Case report			
	The concept of forensic emergency medicine as illustrated by an unusual complication of pulmonary artery catheterization		2008						X		Case report			
misplaced venous catheter AND stent	Successful removal of a central venous catheter misplaced in the right subclavian artery using an intravascular stent: a case report	Yoshida et al.	2021	10.1186/s40981-021-00418-y	X						Case report			X
	Prevention and treatment of dilator injuries during central venous catheter placement		2019			X			X	X	Clinical study			
	Central venous catheter misplaced in the vertebral artery		2016			X	X				Case report			
	Treatment of inadvertent cervical arterial catheterization: Single-center experience		2022			X	X				Clinical study			
	Misplaced central venous catheter in the vertebral artery: endovascular treatment of foreseen hemorrhage during catheter withdrawal	Akkan et al.	2014	10.5301/jva.5000267	X						Case report		X	
	Erroneous placement of central venous catheters in subclavian artery: Retrieval and successful hemostasis with a femoral closure device		2022			X			X		Case report			
	Minimally invasive catheter procedures to assist complicated pacemaker lead extraction and implantation in the operating room		2011			X				X	Clinical study			
	Endovascular foreign body retrieval		2013			X				X	Clinical study			
	Intracaval misplacement of a double-J ureteral stent		2018			X				X	Case report			
	Accidental central venous catheter cannulation into aberrant arterial anatomy requiring endovascular intervention	Lucas et al.	2023	10.1016/j.jvscit.2023.101164	X						Case report			X
	Angioplasty and stenting of a jugular-carotid fistula resulting from the inadvertent placement of a hemodialysis catheter: case report and review of literature	Wadhwa et al.	2016	10.1111/j.1525-139X.2011.01005.x	X						Case report	X		
	Inadvertent arterial placement of central venous catheter: salvage using endovascular treatment	Shaw et al.	2019	10.1136/bcr-2019-231751	X						Case report			X
	Life-threatening vascular complications after central venous catheter placement	Wicky et al.	2002	10.1007/s003300101018	X						Retrospective study			X
	Remove or not, that is the question: A case report on carotid artery cannulation during indwelling venous hemodialysis catheter	Yen et al.	2015	10.1111/hdi.12297	X						Case report	X		
	Misplacement or migration? Extremely rare case of cardiac migration of a ureteral j stent		2014			X			X	X	Case report			
	Caval migration of a ureteral J-stent after simultaneous ureter and iliac vein perforation during its placement for obstructive pyelonephritis		2009			X			X	X	Case report			
	Percutaneous retrieval of a Strecker stent misplaced during TIPS		1995			X			X	X	Case report			
misplaced venous catheter AND rescue	Percutaneous closure of accidentally subclavian artery catheterization: time to change first line approach?		2022			X			X	X	Clinical study			
misplaced broviac AND balloon guided catheter	X													
misplaced broviac AND stroke	X													
misplaced broviac AND cerebral angiography	X													
misplaced broviac AND ischemia	X													
misplaced broviac AND stent	X													
misplaced broviac AND rescue	X													
misplaced shaldon AND balloon guided catheter	X													
misplaced shaldon AND stroke	X													
misplaced shaldon AND cerebral angiography	X													
misplaced shaldon AND ischemia	X													
misplaced shaldon AND stent	X													
misplaced shaldon AND rescue	X													
misplaced central venous catheter AND balloon guided catheter	X													
misplaced central venous catheter AND stroke	X													
misplaced central venous catheter AND cerebral angiography	X													
misplaced central venous catheter AND ischemia	X													
misplaced central venous catheter AND stent	X													
misplaced central venous catheter AND rescue	X													

**Table 2:** Overview of iatrogenic injuries of supra-aortic arteries, including catheter type used, nature of injury, treatment approach, technical success, complications, and clinical outcomes. Abbreviations: CVC: central venous catheter; RVJI: right vena jugularis interna; RSCA: right subclavian artery; HDC: hemodialysis catheter; RACC: right arteria carotis communis; RAVT: right arteria vertebralis; RACP: right arteria cerebri posterior; ACI: arteria carotis interna.

	Treatment Approach						Technical Succes		Surgical Intervention Required		Postinterventionell Complication				Outcome				Special Points						
Vascular Territory	Manual Pressure	Percutaneous Closure	Stenting	Coiling	Balloon Occlusion	Open Surgicl Repair	Yes	No	Yes	No	Hemorrhage	Occlusion	Stroke	Pseudaneurysm	Favorable	Unfavorable	Resolution of Complications								
			X				X			X					X										
			X				X			X					X										
RAVT + RACP		X			X		X			X				X	X		Complete								
			X				X			X					X				RAVT closure without stroke after MRI-perfusion						
			X				X			X					X										
	X		X				X			X					X										
ACI			X				X			X					X				Duration of misplacement of catheter = 24 days, administration of several medications						
			X		X		X			X					X										
				X			X			X					X				Complete posterior circulation blood supply via LAVT						

## Data Availability

The datasets and images used and/or analyzed during the current study are available from the corresponding author on reasonable request.

## References

[1] Lucas S.J., Bready E., Banks C.A., Gaillard W.F., Beck A.W., Spangler E. (2023). Accidental Central Venous Catheter Cannulation into Aberrant Arterial Anatomy Requiring Endovascular Intervention. J. Vasc. Surg. Cases Innov. Tech..

[2] Broviac J.W., Cole J.J., Scribner B.H. (1973). A Silicone Rubber Atrial Catheter for Prolonged Parenteral Alimentation. Surg. Gynecol. Obs..

[3] Fritsch L.-M., Le M., Elrod J., Wössmann W., Vincent D., Reinshagen K., Boettcher M. (2023). Surgical Experience Affects the Outcome of Central Venous Access Catheter Implantation in Children: A Retrospective Cohort Study. J. Pediatr. Hematol. Oncol..

[4] Ladefoged K., Jarnum S. (1978). Long-Term Parenteral Nutrition. Br. Med. J..

[5] Garonzi C., Zeni F., Tridello G., Giacomazzi A., Castagna A., Esposto M.P., Caddeo G., Pezzella V., Zaccaron A., Bonetti E. (2024). Results of a Long-Term, Prospective Study on Complications of Central Venous Catheter in Pediatric Patients with Hematologic-Oncologic Diseases. Pediatr. Blood Cancer.

[6] Blotte C., Styers J., Zhu H., Channabasappa N., Piper H.G. (2017). A Comparison of Broviac^®^ and Peripherally Inserted Central Catheters in Children with Intestinal Failure. J. Pediatr. Surg..

[7] Wicky S., Meuwly J.-Y., Doenz F., Uské A., Schnyder P., Denys A. (2002). Life-Threatening Vascular Complications after Central Venous Catheter Placement. Eur. Radiol..

[8] Jahromi B.S., Tummala R.P., Levy E.I. (2009). Inadvertent Subclavian Artery Catheter Placement Complicated by Stroke: Endovascular Management and Review. Catheter. Cardiovasc. Interv..

[9] Tanveer Ud Din M., Nasrullah A., Sarma D., Ashraf O., Arshad H. (2021). Amidst COVID-19 Pandemic: The Catastrophic Sequelae of an Inadvertent Carotid Artery Insertion during Central Venous Catheter Placement—A Case Report. J. Community Hosp. Intern. Med. Perspect..

[10] Ito Y., Kojima T., Yamanoi Y., Saito K. (2022). Acute Endovascular Therapy for Iatrogenic Vertebral Artery Injury: A Case Report. J. Neuroendovasc. Ther..

[11] Schoder M., Cejna M., Hölzenbein T., Bischof G., Lomoschitz F., Funovics M., Nöbauer-Huhmann I., Sulzbacher I., Lammer J. (2003). Elective and Emergent Endovascular Treatment of Subclavian Artery Aneurysms and Injuries. J. Endovasc. Ther..

[12] Kohyama T., Fujimaki K., Sasamori H., Tokumine J., Moriyama K., Yorozu T. (2023). Inadvertent Catheter Misplacement into the Subclavian Artery during Ultrasound-Guided Internal Jugular Venous Catheterization: A Case Report. JA Clin. Rep..

[13] Yoshida H., Ikemoto S., Tokinaga Y., Ejiri K., Kawamata T. (2021). Successful Removal of a Central Venous Catheter Misplaced in the Right Subclavian Artery Using an Intravascular Stent: A Case Report. JA Clin. Rep..

[14] Akkan K., Cindil E., Kilic K., Ilgit E., Onal B., Erbas G. (2014). Misplaced Central Venous Catheter in the Vertebral Artery: Endovascular Treatment of Foreseen Hemorrhage during Catheter Withdrawal. J. Vasc. Access.

[15] Wadhwa R., Toms J., Nanda A., Abreo K., Cuellar H. (2012). Angioplasty and Stenting of a Jugular-Carotid Fistula Resulting from the Inadvertent Placement of a Hemodialysis Catheter: Case Report and Review of Literature. Semin. Dial..

[16] Shaw M., Chandrashekhara S.H., Sharma A., Kumar S. (2019). Inadvertent Arterial Placement of Central Venous Catheter: Salvage Using Endovascular Treatment. BMJ Case Rep..

[17] Yen C.-C., Chiu Y.-W., Chen H.-C. (2015). Remove or Not, That Is the Question: A Case Report on Carotid Artery Cannulation during Indwelling Venous Hemodialysis Catheter. Hemodial. Int..

[18] Guilbert M.-C., Elkouri S., Bracco D., Corriveau M.M., Beaudoin N., Dubois M.J., Bruneau L., Blair J.-F. (2008). Arterial Trauma during Central Venous Catheter Insertion: Case Series, Review and Proposed Algorithm. J. Vasc. Surg..

